# 

**Published:** 1881-07

**Authors:** 


SURG GENLS OFFS

Vol. XVII. July 1881. No. 2.

### The Bistoury.

Thad S. Up de Graff, M.D.
EDITOR.

ELMIRA, N.Y.

